# Excisional biopsy of perforated gastric ulcer: mandatory or potentially harmful?

**DOI:** 10.1007/s00423-024-03393-x

**Published:** 2024-07-04

**Authors:** Faruk Koca, Christine Koch, Falko Schulze, Ursula Pession, Wolf O. Bechstein, Patrizia Malkomes

**Affiliations:** 1 Department of General, Visceral, Transplant and Thoracic Surgery, Goethe University Frankfurt, University Hospital, Theodor-Stern-Kai 7, 60590 Frankfurt am Main, Germany; 2 Department of Gastroenterology, Hepatology, Endocrinology and Nutrition, Goethe University Frankfurt, University Hospital, Frankfurt am Main, Germany; 3 Senckenberg Institute of Pathology, Goethe University Frankfurt, University Hospital, Frankfurt am Main, Germany

**Keywords:** Peptic ulcer, Spontaneous gastric perforation, Ulcer excision, Gastric cancer

## Abstract

**Purpose:**

This study aimed to evaluate the morbidity associated with excisional biopsy in patients with spontaneous gastric perforation.

**Methods:**

A retrospective, single-center, observational study was performed. All consecutive patients with spontaneous gastric perforation who underwent surgical therapy were included. Outcomes were assessed concerning the performance of excisional biopsy.

**Results:**

A total of 135 adult patients were enrolled. Of these, 110 (81.5%) patients underwent excisional biopsy, while 17 (12.6%) did not. The remaining eight (5.9%) patients who underwent gastric resection were excluded from the analysis. Patients undergoing excisional biopsy developed significantly higher rates of postoperative complications (*p* = 0.007) and experienced more severe complications according to the Clavien-Dindo classification, particularly type III and above (*p* = 0.017). However, no significant differences were observed regarding in-hospital mortality, reoperation, suture dehiscence, or length of hospital stay.

**Conclusion:**

Excisional biopsy for gastric perforation has been shown to be associated with increased morbidity. Surgical closure followed by early endoscopic biopsy may be a superior approach for gastric perforation management to rule out malignancy.

## Introduction

Gastroduodenal ulcers arise from acid-peptic injury to the mucosa of the gastroduodenal tract, with causal factors including *Helicobacter pylori* infection, non-steroidal anti-inflammatory drugs, dietary factors, stress, alcohol and tobacco abuse [1]. Gastroduodenal perforations are associated with significant morbidity and with mortality rates reaching up to 25% [2]. Among perforations, those of the stomach predominate, comprising 74% of cases [2]. It has been reported that gastric cancer accounts for approximately 10–16% of all gastric perforations [3, 4]. Consequently, former studies have recommended biopsy [5] or resection of gastric ulcer perforations, especially those ≥ 2 cm in diameter, to rule out malignancy [6]. However, with a decreasing incidence of gastric cancer, a lower prevalence of malignancy of spontaneous gastric perforation may be observed than reported in the literature. The hypothesis is that in an emergency setting with peritonitis, an intraoperative excisional biopsy to rule out malignancy could be omitted in the context of damage control surgery. A recent prospective observational study suggests that biopsy may be more appropriately conducted endoscopically following recovery from gastric perforation [7]. However, the literature does not address the question of potential adverse consequences of performing excisional biopsies in gastric perforation cases. Therefore, this study aimed to evaluate the incidence of malignancy and outcomes associated with excisional biopsy, considering the diameter of the perforation in gastric ulcer perforation, to assess the necessity of excisional biopsy.

## Materials and methods

This retrospective, single-center, observational study has been approved by the local ethics committee of the Faculty of Medicine at the Goethe University Frankfurt, Germany (reference number: 2024 − 1695). All adult patients with spontaneous gastric perforation, who underwent surgical therapy at the University Hospital Frankfurt from 2002 to February 2024, were included. Data were collected retrospectively from the electronic patients’ records.

### Patients- and procedure-related parameters

Demographic parameters (age at surgery, sex), presence of pre-existing conditions, and medications were recorded.

The location of the perforation was classified as follows: pyloric, prepyloric, antral, or body. The diameter of the perforation was categorized as < 2 cm and ≥ 2 cm. Surgical procedures were classified as follows: primary suture, primary suture with omental patch, and partial gastrectomy with reconstruction. Excisional biopsy involves the removal of a portion of the ulcer at the perforation margin for biopsy. Complete removal of the entire ulcer was not routinely performed. The duration from the onset of abdominal pain associated with gastric perforation to the time of surgical treatment was categorized into 5 groups: up to 6 hours (h), between 6 and 12 h, between 12 and 24 h, between 24 and 72 h, and more than 72 h. Additional parameters recorded included the American Society of Anesthesiologists (ASA) physical status classification [8], postoperative complications according to the Clavien-Dindo classification (CDC) [9], postoperative suture dehiscence, bleeding events, laboratory parameters, and histologic findings of malignancy and *Helicobacter pylori* infection.

### Statistical analysis

Statistical analysis was performed using IBM SPSS version 29.0.0.2 software. Descriptive statistics included median and percentage of total for binary and categorical parameters, as well as median and interquartile range (IQR) for continuous parameters. The Chi-squared test was used to compare excisional biopsy versus no excisional biopsy and perforation diameter < 2 cm versus ≥ 2 cm for the following parameters: age, sex, cardiovascular disease, previous malignancy at the time of surgery, in-hospital mortality, postoperative complications, CDC type ≥ III complications, reoperation, suture dehiscence, and postoperative bleeding. Eight patients who underwent partial gastrectomy were excluded from the analysis. Additionally, seven patients lacking perforation diameter data were excluded from the analysis for the perforation diameter. For comparison of age, ASA classification, the duration from the onset of gastric perforation-related pain to the time of surgery, and length of hospital stay in patients without in-hospital mortality, the Mann-Whitney U test was used. Multivariate analysis was performed with univariate analysis identifying risk factors for outcome parameters (postoperative complications according to CDC, in-hospital mortality, suture dehiscence, reoperation, operative bleeding, and length of hospital stay). Odds ratio (OR) and 95% confidence interval (CI) were calculated.

## Results

### Baseline characteristics and surgical procedures

A total of 135 patients with spontaneous gastric perforation were identified. Of these 79 (58.5%) were male and 56 (41.5%) were female. The median age at the time of surgery was 57 years (IQR 29).

Patients were classified according to the ASA classification as follows: 31 patients (23%) ASA I, 53 patients (39.3%) ASA II, 37 patients (27.4%) ASA III, 12 patients (8.9%) ASA IV, 1 patient (0.7%) ASA V, and 1 patient (0.7%) was not available due to missing data in the medical record. A history of cardiovascular disease was found in 47 (34.8%) patients, previous malignancy in 17 (12.6%), six (4.4%) patients received chemotherapy, six (4.4%) were on steroids, 16 (11.9%) used non-steroidal anti-inflammatory drugs and 16 (11.9%) were on antiplatelet agents or anticoagulants. There was a history of non-gastrointestinal surgery or intervention up to three months prior to ulcer perforation in 10 patients (7.4%).

Radiologically, free intra-abdominal air was detected in 120 (88.9%) patients, with 81 (60%) identified through computed tomography (CT) scans and 39 (28.9%) through plain abdominal film X-rays. Overall, CT scans were performed in 83 patients (61.5%). The duration from the onset of gastric perforation-related pain to the time of surgery was 6 h or less in 40 patients (29.6%), 6–12 h in 17 patients (12.6%), 12–24 h in 10 patients (7.4%), 24–72 h in 22 patients (16.3%), and 72 h or more in 8 patients (5.9%), unfortunately, 38 patients (28.1%) did not have a complete history for various reasons, so these patients could not be subdivided for this analysis (Table 1).

**Table 1:** Demographic and clinical characteristics of patients with spontaneous gastric perforation

Variables *N* = 135	*N* (%)/median (IQR)
Age at the time of surgery (years)	57 (29)
*Sex*	
Male	79 (58.5)
Female	56 (41.5)
*ASA classification*	
I	31 (23)
II	53 (39.3)
III	37 (27.4)
IV	12 (8.9)
V	1 (0.7)
*Comorbidities*	
Cardiovascular disease	47 (34.8)
Malignancy at the time of surgery	17 (12.6)
*Medication*	
Chemotherapy	6 (4.4)
Steroids	6 (4.4)
Non-steroidal anti-inflammatory drugs	16 (11.9)
Antiplatelet agents or anticoagulants	16 (11.9)
Intervention up to 3 months prior to surgery	10 (7.4)
Clinical signs of peritonitis	130 (96.3)
*Diagnosis*	
Computed tomography	83 (61.5)
Free air on computed tomography	81 (60)
Free air on plain abdominal X-ray	39 (28.9)
*Time from pain onset to surgery*	
≤6 h	40 (29.6)
6–12 h	17 (12.6)
12–24 h	10 (7.4)
24–72 h	22 (16.3)
≥72 h	8 (5.9)

ASA- American Society of Anesthesiologists, h- hours, N- number, IQR- interquartile range

Regarding perforation localization 56 patients (41.5%) had pyloric perforation, 51 (37.8%) had prepyloric, 14 (10.4%) had antral and 14 (10.4%) had perforations in the body of the stomach. Perforation diameter was less than 2 cm in 110 patients (81.5%). Only 17 patients (12.6%) underwent laparoscopic surgery. Primary suture was performed in 58 (43%), primary suture with omental patch in 69 (51.1%), and partial gastrectomy with reconstruction in eight (5.9%) patients. Among patients without gastric resection, excisional biopsy was performed in 110 (81.5%), while 17 (12.6%) did not undergo excisional biopsy.

Histologic examination revealed no malignancy in any of the patients. In the group without excisional biopsy (17 patients) four patients were lost to follow-up and two patients did not return for the scheduled endoscopy, so that gastric malignancy could not be completely excluded in six patients (4.4%). However, gastric malignancy could be excluded in 11 patients. Seven patients (5.2%) were found to have *Helicobacter pylori*-associated gastritis. Additionally, empirical *Helicobacter pylori* eradication therapy was initiated in 39 (28.9%) patients.

The median duration of follow-up was 25 days, with an interquartile range of 730 days.

### Outcome parameters after surgery for gastric perforation

Postoperative morbidity occurred in 58 patients (43%), including suture dehiscence in twelve patients (8.9%), postoperative bleeding in four patients (3%), and surgical site infection in twelve patients (8.9%). A total of 39 patients (28.9%) experienced postoperative complications classified as type III or higher according to the CDC. 17 patients (12.6%) were re-operated, including ten patients with suture dehiscence, four patients with fascial dehiscence, one patient with postoperative bleeding, and two patients with intestinal ischemia in septic shock. 13 patients (9.6%) underwent a local superficial procedure, mostly for surgical site infection. The in-hospital mortality rate was 11.9%. The median length of hospital stay was 9 days with an interquartile range of 6 days (Table 2).

**Table 2:** Descriptive statistics of patients with spontaneous gastric perforation

Variables *N* = 135	*N* (%)/median (IQR)
*Localization*	
Pyloric	56 (41.5)
Prepyloric	51 (37.8)
Antral	14 (10.4)
Body	14 (10.4)
*Perforation diameter*	
<2 cm	110 (81.5)
≥2 cm	16 (11.9)
N/A	9 (6.7)
*Type of Surgery*	
Laparoscopic	17 (12.6)
Open	118 (87.4)
*Surgical Procedure*	
Primary suture	58 (43)
Primary suture with omental patch	69 (51.1)
Partial gastrectomy with reconstruction	8 (5.9)
*Excisional biopsy*	
Yes	110 (81.5)
No	17 (12.6)
Malignancy	0
*Helicobacter pylori*-associated gastritis	7 (5.2)
Empirical *Helicobacter pylori* eradication	39 (28.9)
*Postoperative complications according to CDC*	
0	77 (57)
I	7 (5.2)
II	10 (7.4)
IIIA	13 (9.6)
IIIB	7 (5.2)
IV	5 (3.7)
V	16 (11.9)
Re-operation	17 (12.6)
Suture dehiscence	12 (8.9)
Postoperative bleeding	4 (3)
Length of hospital stay [days]	9 (6)
Follow-up (days)	25 (730)

N- number, IQR- interquartile range, CDC- Clavien-Dindo classification

Comparison between groups revealed a postoperative complication rate of 46.4% in the excisional biopsy group, contrasting with 11.8% in the group without excisional biopsy. In addition, complications classified as type III or higher according to the CDC occurred in 34.5% of patients in the excisional biopsy group, compared to 5.9% in the non-excisional group. Chi-squared test showed that patients who underwent excisional biopsy had significantly higher rates of postoperative complications (*p* = 0.007) and type III or higher CDC complications (*p* = 0.017). However, there were no significant differences between excisional biopsy and no excisional biopsy groups concerning in-hospital mortality, re-operation rate, suture dehiscence, or length of hospital stay. Conversely, perforation diameter of ≥ 2 cm was significantly associated with in-hospital mortality (*p* = 0.011) and type III or higher CDC complications (*p* = 0.019). Regarding patient characteristics such as age, gender, ASA classification, cardiovascular disease, and malignancy at the time of surgery, there were no significant diffenreces between the two groups. In the comparison of perforation size, the time interval was significantly longer in the group with a perforation diameter ≥ 2 cm compared to those with a diameter < 2 cm, *p* = 0.048 (Table 3).

**Table 3:** Outcome of excisional biopsy and perforation diameter in patients with spontaneous gastric perforation

	Excisional biopsy *N* = 127		*p* value	Perforation diameter *N* = 126		*p* value
	*N* (%/IQR)			*N* (%/IQR)		
	yes	no		< 2 cm	≥ 2 cm	
N	110	17		110	16	
Age (years)	57 (28)	56 (40)	0.955	56 (29)	70 (26)	0.054
Sex			0.774			0.395
Male	64 (58.2)	11 (64.7))		64 (58.2)	8 (50)	
Female	46 (41.8)	6 (35.3)		46 (41.2)	8 (50)	
ASA classification			0.658			0.117
I	26 (23.6)	5 (29.4)		27 (24.6)	1 (6.3)	
II	43 (39)	6 (35.3)		41 (37.3)	7 (43.8)	
III	28 (25.5)	5 (29.4)		31 (28.2)	5 (31.3)	
IV	11 (10)	1 (5.9)		9 (8.2)	3 (18.8)	
V	1 (0.9)	0		1 (0.9)	0	
Time from pain onset to surgery			0.128			**0.048**
≤6 h	32 (29.1)	7 (41.2)		36 (32.7)	2 (12.5)	
6–12 h	13 (11.8)	4 (23.5)		16 (14.5)	0	
12–24 h	10 (9.1)	0		10 (9.1)	0	
24–72 h	18 (16.4)	2 (11.8)		16 (14.5)	5 (31.3)	
≥72 h	8 (7.3)	0		5 (4.5)	1 (6.3)	
Cardiovascular disease	39 (35.5)	4 (23.5)	0.331	38 (34.5)	7 (43.8)	0.483
Malignancy at the time of surgery	15 (13.6)	0	0.159	14 (12.7)	3 (18.8)	0.397
In-hospital mortality	14 (12.7)	0	0.119	10 (9)	5 (31.3)	**0.011**
Postoperative complications	51 (46.4)	2 (11.8)	**0.007**	44 (40)	10 (62.5)	0.089
Postoperative complications CDC ≥ 3	38 (34.5)	1 (5.9)	**0.017**	30 (27.3)	9 (56.3)	**0.019**
Reoperation	17 (15.5)	0	0.082	13 (11.8)	2 (12.5)	0.937
Suture dehiscence	12 (10.9)	0	0.150	9 (8.2)	3 (18.8)	0.183
Postoperative bleeding	4 (3.6)	0	0.424	3 (2.7)	1 (6.3)	0.453
Length of hospital stay (days)	8 (7)	9.5 (5)	0.234	9.5 (5)	10 (7)	0.831

ASA- American Society of Anesthesiologists, N- number, IQR- interquartile range

Excisional biopsy (yes vs. no), age (≥67 vs. <67 years), diameter (2 cm vs <2 cm), and ASA classification ≥ III (yes vs. no) were included as risk factors in the multivariate analysis for outcome parameters (postoperative complications according to CDC, in-hospital mortality, suture dehiscence, reoperation, postoperative bleeding, and length of hospital stay). In multivariate analysis, we also showed that excisional biopsy was a risk factor for more severe CDC complications (Mean in points 1.3, 95% CI 0.4–2, *p* = 0.003), CDC ≥ 3 (OR 12, 95% CI 1.4–104, *p* = 0.024), and overall postoperative complications (OR 9.6, 95% CI 1.8-48.3, *p* = 0.008), however, it was not found to be an independent risk factor for the composite of these outcome parameters (p = 0.342). On the other hand, diameter (≥2 cm vs. <2 cm) with p value of 0.014, and ASA classification ≥ III (yes vs. no) with p value of 0.002 were identified as independent risk factors for the composite of these outcome parameters (Table 4).

**Table 4:** Multivariate analysis identifying risk factors of spontaneous gastric perforation for outcome parameters

	**Postoperative complication**		**In-hospital mortality**	
	OR (95% CI)	*p* value	OR (95% CI)	*p* value
Excisional biopsy (yes vs. no)	9.6 (1.8–48.3)	0.008	N/A	0.998
Age (≥67 vs. <67 years)	2.5 (1–6.3)	< 0.001	2.7 (0.7–10.1)	0.136
Diameter (≥2 cm vs. <2 cm)	1.4 (0.7–2.8)	0.405	2.4 (0.9–6.3)	0.07
ASA classification ≥ III (yes vs. no)	4 (1.7–9.7)	0.002	6.6 (1.8–24.5)	0.005
	**Suture dehiscence**		**Reoperation**	
Excisional biopsy (yes vs. no)	N/A	0.998	N/A	0.998
Age (≥67 vs. <67 years)	1.5 (0.4–6.3)	0.551	0.9 (0.3-3.3)	0.938
Diameter (≥2 cm vs. <2 cm)	1 (0.4–3.7)	0.760	1.8 (0.7-4.1)	0.199
ASA classification ≥ III (yes vs. no)	2.3 (0.7-8.2)	0.188	1.9 (0.7-5.6)	0.237
	**Postoperative bleeding**		**CDC ≥ 3**	
Excisional biopsy (yes vs. no)	N/A	1	12 (1.4-104)	0.024
Age (≥67 vs. <67 years)	2.3 (0.3-16.9)	0.420	3.1 (1.2-8.1)	0.017
Diameter (≥2 cm vs. <2 cm)	1 (0.16-6.7)	0.965	1.7 (0.8-3.6)	0.178
ASA classification ≥ III (yes vs. no)	1.3 (0.1-12.2)	0.824	3.7 (1.5-9.3)	0.005
	**CDC** Mean (95% CI) in points p value		**Length of hospital stay**	
			Mean (95% CI) in days	*p* value
Excisional biopsy (yes vs. no)	1.3 (0.4–2)	0.003	4 (0–12)	0.202
Age (≥67 vs. <67 years)	0.8 (0.2–1.5)	0.015	N/A	0.943
Diameter (≥2 cm vs. <2 cm)	0.3 (N/A–0.8)	0.257	2 (N/A–6)	0.238
ASA classification ≥ III (yes vs. no)	1.2 (0.6–1.9)	< 0.001	7 (2–12)	0.007

ASA- American Society of Anesthesiologists, CDC- Clavien-Dindo classification, CI- confidence interval, OR- Odds ratio, N/A- not available

## Discussion

Peptic ulcer perforations, which are predominantly gastric, remain significant clinical entities associated with considerable morbidity and mortality, despite the development of effective acid suppressants and declining rates of *Helicobacter pylori* infection.

While some previous studies have reported that 10–16% of all gastric perforations are attributed to gastric cancer, a more recent prospective observational study of 68 patients found no evidence of histologic malignancy [3, 4, 7]. In our cohort of patients with spontaneous gastric perforation, we did not detect a single case of gastric cancer in intraoperative biopsies over a period of 22 years. Although 12.6% of our patients had a history of cancer at the time of perforation, predominantly non-gastrointestinal cancers, an initial diagnosis of gastric cancer was not established via excisional biopsy. Obviously, the incidence of gastric cancer associated with gastric perforation varies depending on the epidemiology of gastric cancer, and thus is likely to differ across geographic regions. Historically, perforation biopsy in gastric perforations was common practice to rule out gastric cancer, predating the era of endoscopy [10, 11]. The approach aimed to achieve precise excision of the ulcer margin to preserve tissue with a good blood supply [12]. However, guidelines from The World Society of Emergency Surgery do not recommend routine biopsy of gastric perforations [6]. Despite this, there are no studies evaluating the outcomes of biopsy in gastric perforation cases. Therefore, we analyzed the impact of biopsy in our cohort. Our analysis revealed that patients undergoing excisional biopsy showed higher rates of general postoperative complications (*p* = 0.007), particularly of type III or higher complications according to the CDC (*p* = 0.017). Steyn and Karusseit recommend initial closure of the perforation followed by endoscopic biopsy to rule out malignancy in modern surgical practice [7]. To facilitate timely endoscopy, comprehensive patient education is imperative to avoid non-compliance.

Excisional biopsies usually enlarge the size of the defect requiring repair and increase the tension on the suture. Increased ulcer size is reported to be an independent predictor of suture leakage, with a 3.3-fold increase in leakage rate for every 10 mm increase in ulcer size [13]. According to Wang et al. even, an ulcer size of ≥ 25 mm can be used as a guide in surgical practice to predict leakage rate [13]. Similarly, Liu et al. showed that perforation diameter in patient with duodenal perforations was an important risk factor for the development of a postoperative leak [14]. Although our retrospective data did not reveal a significant difference in suture dehiscence rate in relation to perforation diameter and excisional biopsy performance in our retrospective data, a trend towards the need for reoperation was evident (*p* = 0.089). Specifically, in multivariate analysis, diameter (≥2 cm vs. <2 cm) was an independent risk factor for worse outcome with a p-value of 0.014. In addition, we demonstrated an association between perforation diameters of 2 cm or greater and in-hospital mortality as well as postoperative complications of type III or higher according to the CDC. 17 of our patients (12.6%) had a previous malignancy in their medical history, many of them in remission and with non-gastrointestinal malignancy. In many cases, chemotherapy was completed long before the gastric perforation. Otherwise, in the presence of active tumor disease under chemotherapy, immunosuppression would be crucial for perioperative morbidity.

The results of this study support closure without biopsy followed by early endoscopic biopsy in cases of spontaneous gastric perforation. It is essential to schedule patients for endoscopy during their hospitalization and provide detailed information to avoid non-compliance and delayed endoscopy for potential gastric cancer diagnosis. Moreover, urgent closure followed by early endoscopic biopsy is the preferred approach for the management of gastric perforation. To determine the incidence of gastric cancer and long-term outcomes of excisional biopsy in patients with spontaneous gastric perforation, future prospective or multicenter studies with larger patient cohorts are warranted.

## Conclusion

Our retrospective analysis shed new light on the morbidity of excisional biopsy for gastric perforation. We have demonstrated, for the first time, that excisional biopsy was associated with increased morbidity compared to cases where no biopsy is performed. Our findings advocate for surgical closure followed by early endoscopic biopsy as the preferred approach for managing gastric perforation enabling clinicians to rule out malignancy, evaluate *Helicobacter pylori*-associated gastritis, or monitor ulcer healing.

## Data Availability

No datasets were generated or analysed during the current study.
